# Supplementary material for: Sustained-input switches for transcription factors and microRNAs are central building blocks of eukaryotic gene circuits
Source: Genome Biol. 2013 Aug 23;14(8):R85. doi: 10.1186/gb-2013-14-8-r85 (PMC4054853; doi:10.1186/gb-2013-14-8-r85)
Supplement: Additional file 5 — HTML Browsable Motif Output. Zipped folder containing all WaRSwap and FANMOD motif output, viewable in a web browser. [file gb-2013-14-8-r85-S5.ZIP › HTML_browsable_motif_output/WaRSwap_all_tair9/sigs_WSWRSLDraw-2000_all.pvals.heatmaps.html/motif_id_14_011110002_tftype_all_upstream_-3000_0.html]

```
BG_MODEL = Rnew2
MOTIF_ID = 14_011110002
TF_TYPE = all
UPSTREAM = -3000_0


PVals
FN_0.2	FN_0.4	FN_0.6	FN_0.8
dg_60.genes	0.301	0.006	0.017	0.769
dg_70.genes	0.226	0.001	0.014	0.746
dg_80.genes	0.679	0.117	0.134	0.615

ZScores
FN_0.2	FN_0.4	FN_0.6	FN_0.8
dg_60.genes	0.525	2.978	2.754	-0.928
dg_70.genes	0.8	3.349	2.944	-0.876
dg_80.genes	-0.543	1.249	0.961	-0.752

StDevs
FN_0.2	FN_0.4	FN_0.6	FN_0.8
dg_60.genes	115632.727	58636.742	18947.078	3113.017
dg_70.genes	111769.735	55291.408	18216.105	3151.196
dg_80.genes	90729.534	45871.466	16560.718	2436.359
```
